# A Novel In Vivo siRNA Delivery System Specifically Targeting Liver Cells for Protection of ConA-Induced Fulminant Hepatitis

**DOI:** 10.1371/journal.pone.0044138

**Published:** 2012-09-06

**Authors:** Nan Jiang, Xusheng Zhang, Xiufen Zheng, Di Chen, Kingsun Siu, Hongmei Wang, Thomas E. Ichim, Douglas Quan, Vivian McAlister, Guihua Chen, Wei-Ping Min

**Affiliations:** 1 Multi-Organ Transplant Program, London Health Sciences Centre, London, Ontario, Canada; 2 Department of Surgery, Pathology, and Oncology, University of Western Ontario, London, Ontario, Canada; 3 Liver Transplant Center, The Third Affiliated Hospital of Sun Yat-sen University, Transplantation Research Institute of Sun Yat-sen University, Guangzhou, China; 4 Jiangxi Provincial Institute of Medical Science, and Medical School of Nanchang University, Nanchang, China; 5 Medistem Laboratories, San Diego, USA; Duke University, United States of America

## Abstract

**Background:**

Fulminant hepatitis progresses to acute liver failure (ALF) when the extent of hepatocyte death exceeds the liver's regenerative capacity. Although small interfering RNA (siRNA) appears promising in animal models of hepatitis, the approach is limited by drawbacks associated with systemic administration of siRNA. The aim of this study is to develop a hepatocyte-specific delivery system of siRNA for treatment of fulminant hepatitis.

**Methodology/Principal Findings:**

Galactose-conjugated liposome nano-particles (Gal-LipoNP) bearing siRNA was prepared, and the particle size and zeta potential of Gal-LipoNP/siRNA complexes were measured. The distribution, cytotoxicity and gene silence efficiency were studied *in vivo* in a concanavalin A (ConA)-induced hepatitis model. C57BL/6 mice were treated with Gal-LipoNP Fas siRNA by i.v. injection 72 h before ConA challenge, and hepatocyte injury was evaluated using serum alanine transferase (ALT) and aspartate transaminase (AST) levels, as well as liver histopathology and TUNEL-positive hepatocytes. The galactose-ligated liposomes were capable of encapsulating >96% siRNA and exhibited a higher stability than naked siRNA in plasma. Hepatocyte-specific targeting was confirmed by in vivo delivery experiment, in which the majority of Gal-LipoNP-siRNA evaded nuclease digestion and accumulated in the liver as soon as 6 h after administration. In vivo gene silencing was significant in the liver after treatment of Gal-Lipo-siRNA. In the ConA-induced hepatitis model, serum levels of ALT and AST were significantly reduced in mice treated with Gal-lipoNP-siRNA as compared with control mice. Additionally, tissue histopathology and apoptosis showed an overall reduction of injury in the Gal-LipoNP siRNA-treated mice.

**Conclusions/Significance:**

This study is the first to our knowledge to demonstrate reduction of hepatic injury by liver-specific induction of RNA interference using Gal-LipoNP Fas siRNA, highlighting a novel RNAi-based therapeutic potential in many liver diseases.

## Introduction

Fulminant hepatitis is a devastating liver disease caused by autoimmune hepatitis, alcohol consumption, chronic or acute viral hepatitis infection, which remains a significant cause of morbidity and mortality internationally [1], [2]. Fulminant hepatitis is associated with severe hyperbilirubinemia, hepatic encephalopathy and death. However, the underlying pathogenesis mechanisms are not fully elucidated. Therefore, a therapeutic approach is difficult and stringent need new accepted therapy that will ameliorate or prevent fulminant hepatitis.

Induction of RNA interference (RNAi) using short interfering RNA (siRNA) promises superior advantages to other drug-development approaches given easy of design, high target selectivity, and expected low toxicity due to metabolism to natural nucleotide components by the endogenous cell systems [3]–[7]. However, siRNA therapeutics is hindered by the poor intracellular uptake and limited blood stability of siRNA which limits effective delivery of the therapeutic molecules to in vivo targets. When siRNA is administered intravenously, it is readily digested by nucleases and largely cleared from the kidney glomeruli before reaching the diseased organs. In order to facilitate site-specific delivery and achieve the therapeutic effect, it is important to increase the stability of siRNA duplexes in the blood circulation and enable the targeted delivery directly to the diseased area.

In vivo delivery techniques that have been applied to siRNA have historically included viral vectors [8], hydrodynamic injection [9] and cationic liposomes [10]. However, while viral vector-based siRNA delivery overcomes the problem of poor transfection, clinical application of this approach is beset by concerns about potential adverse effects such as oncogenesis [11]–[13]. Previous studies have showed that hydrodynamic injection of naked siRNA is effective in reducing the expression of Caspase 8 [14] or Fas [9] in mouse fulminant hepatitis model and reduces corresponding gene mediated liver injury. Although this method can reach the desired gene silencing effect, drawbacks exist such as non-selective delivery of the nucleotides, and clinical impracticality due to need for large volume and high injection speed [15]. In recent years, cationic liposomes emerged as the most promising delivery system due to ability to protect from circulating nucleases and escape from intracellular endosomal degradation [16]–[18].

In this study, we report a novel liver-specific targeting method for siRNA delivery; the use of the galactose-guided liposomal-based siRNA delivery system is efficient for silencing Fas, a critical mediator of hepatocyte death. We demonstrate effective gene silencing through local delivery of the siRNA, and prevention of hepatic injury using this selective delivery system.

## Materials and Methods

### Chemicals

1,3-Dioleoyl-3-trimethylammonium propane (DOTAP), cholesterol, 1,2-Dipalmitoyl-sn-Glycero-3-Phosphocholine (DPPC), 1,2-distearoyl-*sn*-glycero-3-phosphoethanolamine-N-[carboxy(polyethylene glycol)-2000] (DSPE-PEG_2000_-Carboxylic Acid) and 1,2-disteraoyl-sn-glycero-3-phosphoethanolamine-N-[methoxy(polyethylene glycol)-2000] (DSPE-PEG_2000_) were purchased from Avanti Lipids, Inc. (Alabaster, AL). 4-Aminophenyl-D-galactopyranoside, N-(3-Dimethylaminopropyl)-N′-ethylcarbodiimide hydrochloride (EDAC) and Protamine sulfate purchased from Sigma. All other chemicals were of reagent grade.

### Mice

Male, 6–8 week old C57BL/6 mice were purchased from Jackson Laboratory (Bar Harbor, ME, USA). The mice were maintained under specific pathogen-free conditions. All experiments were performed in accordance with the Guide for the *Care and Use on Animals Committee Guidelines*. The Institutional Animal Use subCommittee (AUS) at University of Western Ontario approved all procedures.

### Fas-siRNA design

siRNA with 19-nt length, targeting Fas gene was designed according to our previous report [19] and synthesized by Dharmacon Inc (Lafayette, Colo). The sequence of Fas siRNA was 5′-GUGCAAGUGCAAACCAGAC-3′.

### Synthesis DSPE-PEG_2000_- Galactose lipid

DSPE-PEG_2000_-Galactose lipid was prepared according to the method reported previously [20]. DSPE-PEG_2000_-Carboxylic Acid (0.12 mmol) lyophilized and then dissolved in DMSO (2 mL) solution and mixed with EDAC(0.6 mmol) incubation at room temperature for 10 min, the mixture was supplemented with an excess amount of 4-Aminophenyl D-galactopyranoside (0.24 mmol) and pyridine (2 ml) incubated at room temperature overnight. Pyridine was removed by rotary evaporation. After the reaction mixture was evaporated in vacuo, the resultant material was suspended in water, and dialyzed against distilled water for 48 h using a 3 kDa cut-off membrane (Spectrum, USA). After the dialysis, the products were centrifuged, filtered with Amicon Ultra-15 centrifugal filter units (Millipore; MWCO 3KDa) and freeze-dried.

### Preparation of siRNA

A total of 50 µg Cy3-labeled Fas siRNAs were mixed with full-length recombinant protamine in a 1∶5 (siRNA∶protamine) molar ratio, in DNA RNAase nuclease-free water (Gibco-Invitrogen) and were pre-incubated for 20 min at room temperature to form a complex.

### siRNA-liposome preparation

Multilamellar liposomes, composed of 1,2-Dioleoyl-3-Trimethylammonium -Propane (DOTAP), 1,2-Dipalmitoyl-sn-Glycero-3-Phosphocholine (DPPC) and cholesterol(Chol) (Avanti Polar Lipids, Alabaster, AL, USA) at molar ratios of 2∶7∶7 (DOTAP/DPPC/Chol) were prepared by a thin lipid film method. Briefly, the lipids were dissolved in chloroform in a glass tube and gently dried under nitrogen and further evaporated to dryness under vacuum. The lipid film was hydrated with a swelling solution composed of protamine and the condensed siRNA (5 nmol) was dissolved in RNase nuclease-free water to create multilamellar liposomes. The resulting multilamellar liposomes suspension was exposed to 5 freeze/thaw cycles by alternately placing the sample vial in a dry ice bath and warm water bath. The dispersion was then vortexed for 5 min and sonicated for 2 min using a bath sonicator above the Tc(50°C) of the lipid and then subjected to extrusion (Avanti Polar Lipids, USA) through polycarbonate membranes (Avanti Polar Lipids, USA) with gradually decreasing pore size (0.4, 0.2 and 0.1 mm) to produce unilamellar nano-particle (NP) with 30 cycles each at 50°C.

Targeting liposome (Gal-LipoNP) and non-targeting liposome (LipoNP) were generated by incubating the mixture of unilamellar nanoscale liposome suspension with 10 mol% micelle solution of Gal-DSPE-PEG2000, DSPE-PEG2000, respectively, at 50°C for 10 min, and then cooled down at room temperature.

### Particle diameter and zeta potential measured

The Non-targeting and targeting liposome were freshly prepared and diluted with phosphate-buffered saline (PBS), and the mean particle diameter and surface charge (zeta potential) measured by dynamic light scattering and Malvern Zetasizer nano ZS (Malvern Instruments Ltd., Worcestershire, UK),according to the manufacturers' protocols.

### Efficiency of siRNA entrapment inside liposomes

The siRNA entrapment efficiencies were determined using Cy3-labeled siRNAs. Samples were diluted in nuclease free water to the concentration that falls within the linear range of the standard curve. The sample concentrations were then calculated accordingly to standard curve. The intensity of siRNA encapsulated within liposomes was measured fluorescence intensities in the presence or absence of Triton X-100 at wavelengths of excitation 544 nm and emission 590 nm, which destabilized lipid particles and allowed the release of entrapped siRNA.

### Protection of siRNA by liposomes in mouse plasma

To assess the stability of liposomes-siRNA complex in blood plasma in vitro, 30 µL siRNA (2 µg) was incubated with 270 µL fresh mouse plasma at 37°C for various time periods.The siRNA were either naked or encapsulated by PEGylated liposome. The siRNA inside liposomes were extracted by 0.1% Triton X-100 solution. The reaction was terminated with 10% sodium dodecyl sulfate solution. The degradation of liposome-siRNA complex was determined by 12% polyacrylamide gel electrophoresis followed by visualization by a Fluor-S Multi-Mager (Bio-Rad).

### In vivo biodistribution of liposomes/siRNA complex

Cy3-labeled siRNA(50 µg) was encapsulated by Gal-LipoNP and LipoNP. Mice were treated with liposomes/siRNA complex via tail vein. Other control groups included injection of same amount of naked Cy3-labeled siRNA and naive group. At 6 h, 24 h and 48 h following administration, the organs including heart, liver, spleen, lung and kidney were harvested and rinsed with saline, frozen in liquid nitrogen and mounted for cryostat sectioning. Slides were viewed under a fluorescence microscope.

### Fluorescence intensity and gene silencing efficiency

Different doses of Fas-siRNA (0.125 nmol/g∼0.75 nmol/g) were encapsulated by Gal-LipoNP. Mice were treated with liposomes/siRNA complex via tail vein injection. 6 h later, livers were harvested and rinsed with saline, frozen in liquid nitrogen and mounted for cryostat sectioning. Slides were viewed under a fluorescence microscope. As a parallel experiment, another group of mice were exposed to the same treatment. 72 h later, the expression of Fas gene in liver was detected by quantitative PCR.

### Detection of non-specific inflammation in liver

Three groups of mice were injected with Gal-LipoNP/Fas siRNA(0.25 nmol/g) complex,Gal-LipoNP/Gl2 siRNA(0.25 nmol/g) complex and PBS via tail vein respectively. 4 h later, the livers were extirpated and total RNA was extracted, expression levels of IFN-γ, TNF-α and IL-6 mRNA were detected by real-time PCR.

### Fulminant hepatitis model and siRNA treatment

ConA (Sigma) was dissolved in pyrogen-free PBS at a concentration of 1 mg/ml and injected intravenously through the tail vein (15 mg/kg) [21]. To investigate the efficacy of protection on fulminant hepatitis injury by siRNA, the mice were administrated with siRNA 72 h before ConA injection. 0.25 nmol/g of Fas siRNA were encapsulated by Gal-LipoNP and LipoNP, then diluted in 300 µl of PBS and injected into mice at a normal pressure by tail vein. Hydrodynamic administration was performed by intravenous injection of naked siRNA in a volume of 1.6 ml. Control groups included Gal-LipoNP/Gl2 siRNA and PBS-treated mice.

### Assessment of liver function

Blood samples were obtained from the inferior vena cava at 24 h after ConA injection. Serum levels of aspartate aminotransferase (AST) and alanine aminotransferase (ALT) were measured by the core laboratory at the London Health Sciences Center to monitor liver function.

### Liver histology and apoptosis

At 24 h post- ConA injection, livers were dissected from mice and tissue slices were fixed in 10% formalin and processed for histology examination using standard techniques. Formalin tissue was embedded in paraffin and 5 µm sections were stained with H&E. Ten random fields were assessed for necrosis by standard morphologic criteria. These sections were examined in a blinded fashion by a pathologist. For analysis of apoptosis, TUNEL assay was performed. Liver tissue sections were stained with the in situ cell-death detection kit (Roche) following the manufacturer's instructions. TUNEL-positive cells were determined by observing 1000 cells in randomly selected fields [22].

### Quantitative PCR

Total RNA was extracted from livers using Trizol (Invitrogen). RNA was reverse-transcribed using oligo-(dT) primer and reverse transcriptase (Invitrogen). Quantitative PCR reactions were performed to examine gene expression in Stratagene MX 4000 multiplex quantitative QPCR system using SYBR Green PCR Master mix (Stratagene, La Jolla, CA, USA) and 100 nM of gene-specific forward and reverse primers. Primers used for the amplification of murine Fas, TNF-α, IFN-γ, IL-6 and GAPDH were as follows: Fas, 5′- AAGGGAAGGAGTACATGGACAAGA -3′ (forward) and 5′- GTATGGTTTCACGACTGGAGGTTC -3(reverse); TNF-α, 5′-CTCCCTCCAGAAAAGACACCAT-3′(forward); IFN-γ, 5′-CCATCGGCTGACCTAGAGAAGACA-3′(forward) and 5′-AGCCAGAAACAGCCATGAGGAAGA-3′(reverse);IL-6, 5′-CAACCACGGCCTTCCCTACT-3′(forward) and 5′-TCATTTCCACGATTTCCCAGAG-3′(reverse) and GAPDH, 5′-TGATGACATCAAGAAGGTGGTGAA-3′(forward) and 5′ -TGGGATGGAAATTGTGAGGGAGAT-3′ (reverse). The reaction conditions were 10 min at 95°C, 15 s at 95°C, 1 min at 58°C and 1 min at 72°C (40 cycles). Samples were normalized using the housekeeping gene GAPDH, and a comparative CT method was used for the analysis.

### Western Blot

Liver tissue samples were lysed in ice-cold RIPA buffer (Cell Signaling Technology, Danvers MA) supplemented with 1 mM phenylmethanesulfonylfluoride (PMSF, Sigma) on ice for 15 min. Insoluble material was removed by subsequent centrifugation at 14,000 rpm for 10 min at 4°C. Protein concentrations were determined by the Bradford assay (Bio-Rad, Hercules, CA). Fifty micrograms of protein was size fractionated using SDS-PAGE and transferred to nitrocellulose membranes (Bio-Rad). The membranes were blocked by 5% non-fat milk containing 1X Tris-buffered saline and 0.1% Tween20 (TBST) for 1 h at room temperature, and then incubated with specific antibodies specific for Fas and β-actin (Santa Cruz, CA, USA) at 4°C overnight. Membranes were subsequently washed with TBST and then incubated with the horseradish peroxidase-conjugated secondary antibodies (Santa Cruz) for 1 h at room temperature to. Finally, the membranes were washed and ECL signal detection kit was used (Amersham, IL, USA). The signal was detected by Fluorchem System (ProteinSimple, Santa Clara, CA).

### Statistical analysis

All results are presented as means ± SD. Statistical comparisons between groups were performed using Student's t-test. Statistical significance was determined as p<0.05.

## Results

### Formulation and characterization of Gal-LipoNP

In this study, Fas siRNA was encapsulated within the aqueous interior of Lipo-NP that were conjugated to the galactose, a ligand that specifically binds the hepatocyte-specific asialoglycoprotein receptor (ASGP-R) [23]. Gal-LipoNP were prepared by first mixing Cy3-labeled Fas siRNA and protamine with liposomes composed of the neutral lipid cholesterol, DPPC and cationic lipid DOTAP to form liposomes through the freeze/thawing technique. Subsequently, liposomes were repeatedly extruded through polycarbonate filter membranes with pore sizes of 400, 200, 100 nm to produce a liposome population of uniform size. Subsequently, the post insert technique [24] was used to allow galactose lipids modify surface of the cationic liposomes complex to construct liposome-based targeted NPs for siRNA delivery.

The physicochemical characteristics of the liposome/siRNA complex are summarized in Table 1. The particle diameters of targeting liposome (Gal-LipoNP) and non-targeting liposome (LipoNP) were similar (115.9±0.46 nm vs 120.2±0.87 nm), whereas without DSPE-PEG lipid modified siRNA liposome were 10∼15 nm smaller than both of them. The zeta potential was reduced by DSPE-PEG surface modification (14.17±1.38 mV and 12.67±1.58 mV) compared with those of naked siRNA liposome (24.23±0.67 mV), suggesting that electrostatic interaction between PEG, liposomes and siRNA controlled the size and magnitude of the zeta potential.

**Table 1:** Particle size, zeta potential and envelopment efficiency of liposomes/siRNA complex.

Formulations	Particle size (nm)	Potential (mV)	Envelopment efficiency (%)
Liposomes/Fas siRNA	106.63±1.11	24.23±0.67	96.7±1.4
DSPE-PEG Liposomes/Fas siRNA	120.2±0.87	12.67±1.58	94.9±1.1
Gal-DSPE-PEG Liposomes/Fas siRNA	115.9±0.46	14.17±1.38	95.2±1.9

### Stability of liposome/siRNA complex in mouse plasma

**Figure 1:**
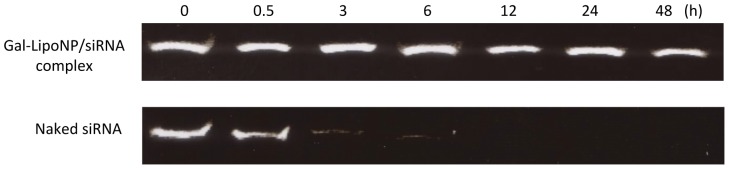
Stability of galactosylated liposomes/siRNA complex in mouse plasma. Cy3-labeled siRNA was complexed with galactosylated liposomes or naked siRNA and was subsequently incubated with fresh mouse plasma at 37°C for various time periods. After incubation for 0, 1, 3, 6,12, 24, and 48 h, siRNA from galactosylated liposomes was extracted with Triton X-100 and detected by polyacrylamide gel electrophoresis.

To determine the stability of the liposome/siRNA complex, liposome/siRNA complex and naked siRNA were exposed to mouse plasma that is known to contain numerous RNA-degrading activities, such as various RNases. Naked siRNA degradation was observed after 30 min of incubation with plasma and almost more than 90% digestion by nucleases was observed within 6 h. In contrast, liposome/siRNA complex displayed significant stability under the same conditions with no significant degradation detected at the 48 h (Figure 1). Taken together, these data suggest that the liposome exerts a protective effect on the siRNA, thus supporting in vivo application.

### Distribution of Gal-LipoNP in vivo

**Figure 2:**
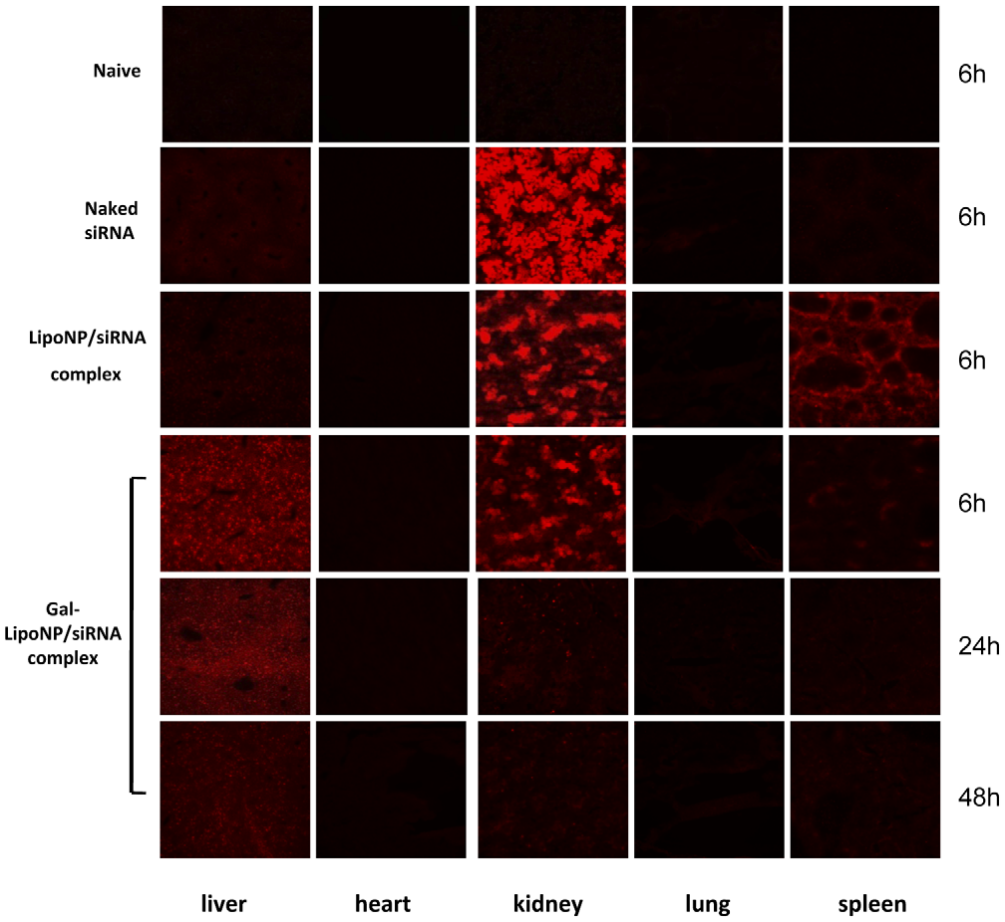
Distribution of liposomes/siRNA complex *in vivo*. C56BL/6 mice were i.v. injected with naked Cy3-labeled siRNA (Naked siRNA), non-targeted liposomes (lipoNP/siRNA) and targeted liposomes with Cy3-labeled siRNA complex (Gal-LioNP/siRNA). Untreated mice served as naïve control. At 6 h, 24 h and 48 h following administration, mice were sacrificed and heart, liver, spleen, lung, kidney were collected. The remaining siRNAs in organs were detected by fluorescence microscope.

In order to test whether Gal-LipoNP can specifically target hepatic tissue, we investigated the biodistribution of siRNA *in vivo* at 6 h, 24 h, 48 h after administration of the galactosylated liposomes/Cy3-labeled siRNA complex. Figure 2 shows images of frozen sections of different organs at the 6 h time point under fluorescence microscopy. In the Gal-LipoNP/siRNA complex injected mice, Cy3 labeled siRNA was detected abundantly in liver and the fluorescence intensity was higher than that in the mice injected with LipoNP/siRNA complex. In contrast, animals treated with control LipoNP/siRNA displayed significant accumulation in the spleen and only small amounts of siRNA in liver cells. Most of naked siRNA was eliminated from the blood and excreted from kidney. Additionally, Gal-LipoNP prolonged the circulation of siRNA in the mouse bloodstream compared with naked siRNA; the fluorescence was still detectable in the liver 24 h and 48 h after injection of Gal-LipoNP/siRNA complex. These data suggest the possibility that Gal-LipoNP/siRNA slowly distributed in organs instead of eliminated in short time.

### Fas expression can be silenced by Gal-LipoNP in vivo

**Figure 3:**
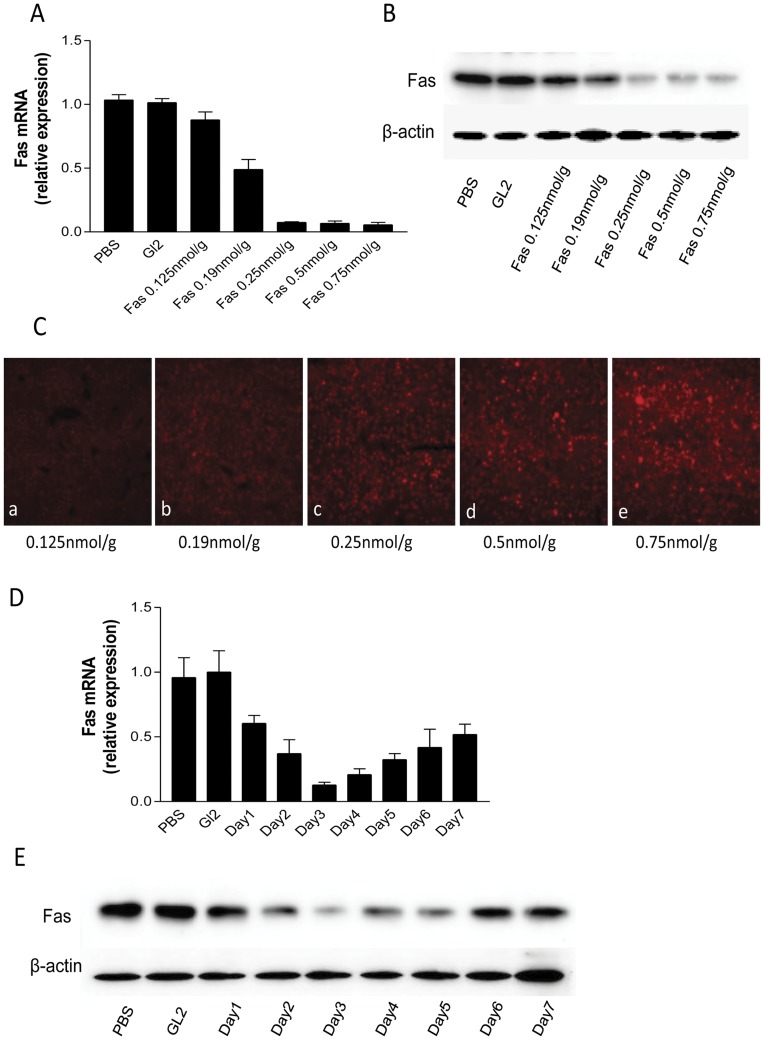
Silencing Fas *in vivo*. ***(A&B) Effect of siRNA dose on gene silencing in mice***. Gal-liposomes/Fas-siRNA complex was i.v. injected into C57BL/6 mice (n = 6, per group) at a siRNA dose from 0.125 nmol/g to 0.75 nmol/g. Gl2 siRNA and PBS treatments were used as negative controls. Gene silencing of Fas in liver was determined using quantitative RT-PCR (A) and western blot (B). ***(C) fluorescence intensity***. Mice were treated with Cy3-labeled Gal-liposomes/Fas-siRNA complex as described in (A). The liver tissues were sectioned and the fluorescence was detected under microscope as described in Materials and Methods. ***(D&E) Dynamic observation of gene silencing***. Gal-liposomes/Fas-siRNA complex was i.v. injected into C57BL/6 mice at a siRNA dose of 0.25 nmol/g (n = 6, per group). Gl2 siRNA and PBS treatments were used as negative controls (n = 6, per group). Gene silencing in liver in different time point was determined using quantitative RT-PCR (D) and western blot (E).

Upon confirmation that siRNA can be delivered to liver tissue through systemic administration of galactosylated liposome, we next investigated the ability of the siRNA suppresses endogenous gene expression in liver. In this study, we chose Fas as a sample target gene, given that recent evidence has implicated Fas highly expressed on hepatocytes and causes hepatic injury through Fas-mediated apoptosis [25], [26]. We first tested the gene silence efficiency in different doses. Mice were treated with different dose Fas-siRNA encapsulated by Gal-LipoNP (0.125 nmol/g∼0.75 nmol/g) via tail vein. 72 h later, the expression of Fas gene was detected by real-time PCR and western blot (Figure 3A and 3B). We found that Gal-LipoNP/Fas-siRNA complex induces silencing rate and the fluorescence intensity have dose-dependent manner. When 0.125 nmol/g siRNA was administered to mice, only about 13% gene silencing effect was observed; 0.19 nmol/g siRNA was administered to mice, about 50% gene silencing effect was observed. When increase siRNA dose from 0.25 nmol/g to 0.75 nmol/g, gene silencing effect can reach above 90%, however, suppression of Fas gene expression was not different between them (Figure 3A and 3B). Figure 3C shows the fluorescence intensity increase with the siRNA dose from 0.25 nmol/g to 0.75 nmol/g. The results indicate that the effective range of siRNA dose-dependence is from 0.25 to 0.75 nmol/g when Gal-LipoNP is used. Based on these results, we choose 0.25 nmol/g as treatment dose in later experiments.

We subsequently investigated the time course of gene-inhibition achieved by Gal-LipoNP. Mice were treated with Gal-LipoNP/Fas siRNA (0.25 nmol/g) complex via tail vein and subsequently sacrificed mice daily from day 1 to day 7 to assays Fas gene expression by quantitative PCR and western blot. We found that Gal-LipoNP/Fas siRNA complex induces silencing efficiency reach peak at the third day and then gradually attenuated, but at the seventh day, 50% gene silencing effect was observed (Figure 3D and 3E). Based on these results, we choose administered Gal-LipoNP/Fas siRNA complex three days before ConA-induced fulminant hepatitis.

### Gal-LipoNP/siRNA complex does not induces non-specific inflammatory responses in liver

**Figure 4:**
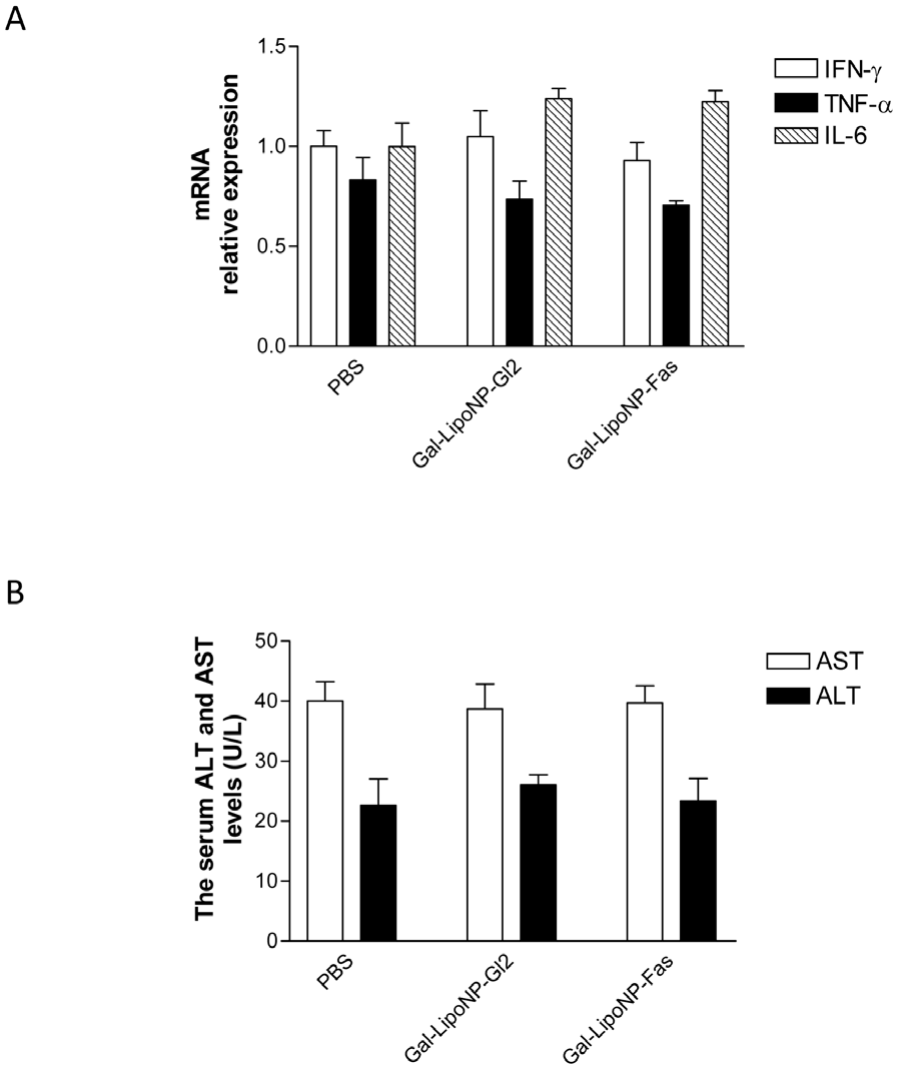
Non-specific inflammatory response after injection of siRNA/liposome complex. ***(A) Liver IFN-γ, TNF-α and IL-6 mRNA levels after administration of galactosylated liposomes/siRNA complex in mice***. Groups of three mice were i.v. injected with Gal-LipoNP complexed with Fas siRNA, Gl2 siRNA and PBS, respectively. At 4 h following administration, liver tissue were collected, IFN-γ, TNF-α and IL-6 levels were measured by quantitative PCR. ***(B)Serum ALT and AST levels after administration of galactosylated liposomes/siRNA complex***. Groups of three mice were i.v. injected with Gal-LipoNP complexed with Fas siRNA, Gl2 siRNA and PBS, respectively. Blood samples were collected at 24 h following administration of experimental agents. AST and ALT levels were measured as described in Materials and Methods.

Previous siRNA approaches have been associated with non-specific inflammatory responses [27], which hinders therapeutic development. To determine whether such responses may occur using our new approach, we assessed hepatic levels of IFN-γ, TNF-α as well as IL-6 using real-time PCR at 4 h following administration Gal-LipoNP/siRNA complex. As shown in Figure 4A, the level of these inflammatory cytokines induced by Gal-LipoNP/Fas siRNA and control Gal-LipoNP/Gl2 siRNA were not higher than that in PBS controls. The Gal-LipoNP/siRNA complex did not increase ALT and AST levels in mouse serum compared with the levels in PBS treated mice (Figure 4B). These results indicate that administration of Gal-LipoNP/siRNA complex do not induced non-specific inflammatory responses and hepatocyte injury.

### Gal-LipoNP/siRNA complex attenuates ConA-induced liver dysfunction

**Figure 5:**
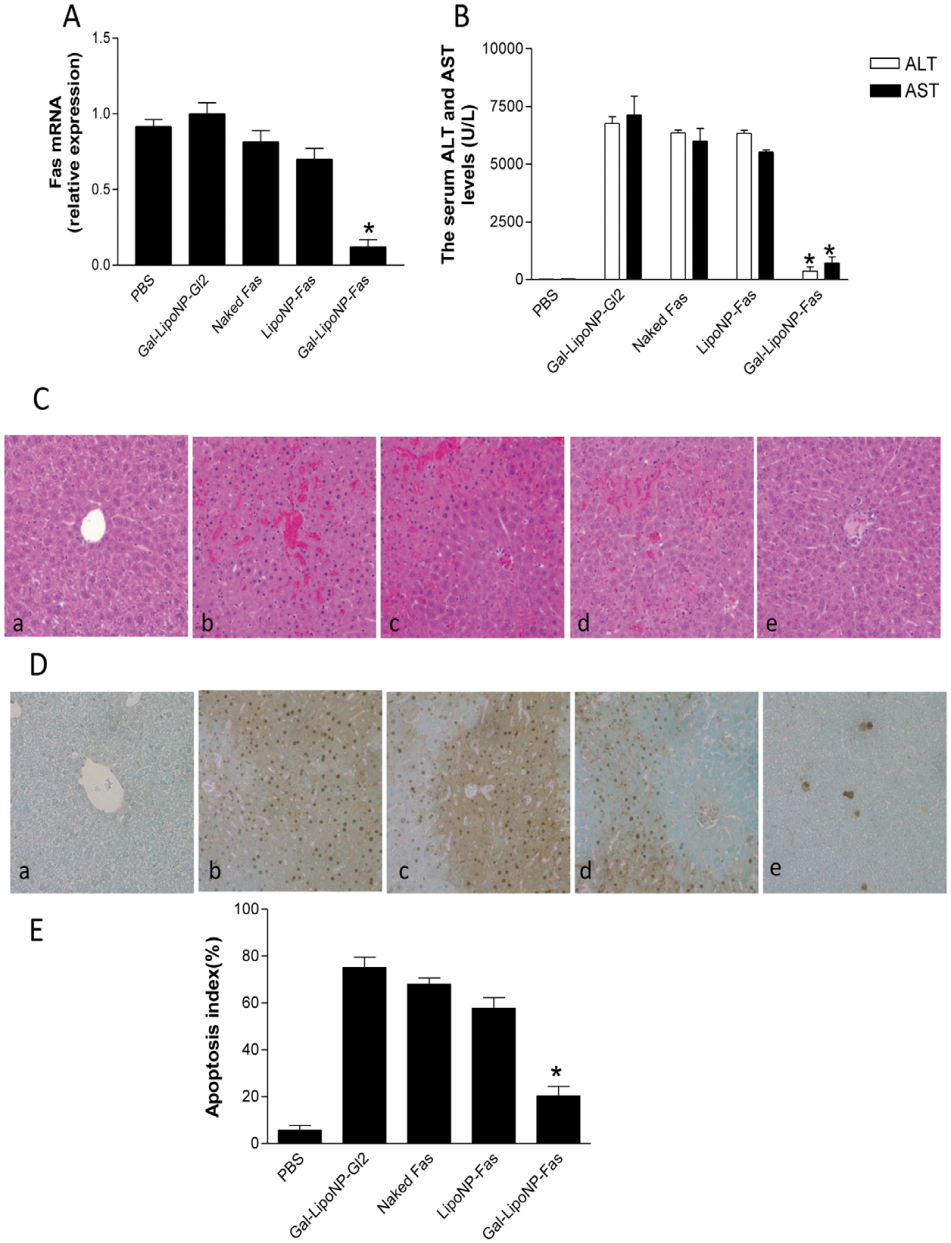
Protecting ConA-induced hepatic injury using Gal-LipoNP/Fas siRNA. C57BL/6 mice were treated with Gal-LipoNP containing Fas siRNA or GL2 siRNA, LipoNP that incorporated with Fas siRNA, Other control groups included naked siRNA administration by hydrodynamic injection and PBS group. 72 h after gene silencing, mice were administered (15 mg/kg) ConA intravenously. After 24 h injury, liver tissues were collected and total RNAs were extracted. Fas Gene suppression (A) and ALT and AST levels (B) were measured. The livers were stained with H&E (C). The apoptotic cells in the liver tissues were detected using TUNEL assay (D), and the percentage of apoptotic cells were counted (E). (a) C57BL/6 mice treated with PBS; (b) Gal-LipoNP containing GL2 siRNA; (c) naked Fas siRNA administration by hydrodynamic injection ; (d) LipoNP containing Fas siRNA; (e) Gal-LipoNP containing Fas siRNA. Values are given as mean ± SD (n = 6, per group). (*) Statistical significance when compared with Gal-LipoNP/Fas siRNA and Gal-LipoNP/GL2 siRNA groups was denoted at p<0.05.

We used ConA induced fulminant hepatitis model to compare expression of Fas mRNA in different treatment groups. The siRNA treatments were performed 72 h before ConA challenge. We administered 0.25 nmol/g Fas-siRNA encapsulated by Gal-LipoNP and LipoNP via tail vein treated mice. The gene suppression effects were also compared with those following hydrodynamic injection of naked Fas-siRNA. Other controls included use scrambled sequence Gl2 siRNA were encapsulated by Gal-LipoNP and PBS groups. The silencing effect of the Fas gene expression in liver was observed with each complex after Fas-siRNA administration. The Gal-LipoNP/Fas siRNA group exhibited greater suppression of Fas gene expression compared with LipoNP/Fas siRNA, as well as the Gal-LipoNP/Gl2 siRNA and naked Fas siRNA treated groups. The gene silencing efficiency only less than 20% in hydrodynamic injection delivery naked Fas siRNA group (Figure 5A). These results show that the Gal-LipoNP exhibited effective means as a hepatic targeting delivery system for siRNA gene silencing in vivo.

Given that siRNA appeared to block the expression of Fas, we hypothesized that siRNA also would prevent liver injury caused by Fas mediated hepatic injury. Accordingly, we measured ALT and AST levels 24 h after ConA injection, to assess the degree of liver dysfunction, the levels of both ALT and AST in Gal-LipoNP Gl2 siRNA, naked Fas siRNA and LipoNP Fas siRNA treated group significantly increased when compared with PBS group. However, treatment with Gal-LipoNP/siRNA complex, prior to inducing hepatitis, significantly prevented increase of ALT and AST values 24 h after hepatocyte injury (Figure 5B).

### Gal-LipoNP/siRNA complex prevents ConA-induced inflammatory response and apoptosis

Histopathology changes and apoptotic hepatocytes of hepatitis injury, as an additional indicator of tissue injury were subsequently examined as an additional measure of protection from ConA-induced pathology. As seen in Figures 5, control groups (Gal-LipoNP Gl2 siRNA, naked Fas siRNA and LipoNP Fas siRNA treated mice) showed evident edema, hepatocellular necrosis, neutrophil invasion (Figure 5C) and high numbers of TUNEL-positive hepatocytes (Figure 5D and 5E). In contrast, mice pretreated with Gal-LipoNP Fas-siRNA demonstrated significant attenuation of all pathological changes and marked reduction in cell apoptosis.

## Discussion

Chronic or acute hepatitis B and C virus infection often progresses towards fulminant hepatitis, which is associated with high level of morbidity and mortality, which represents a major unmet medical need. Given the known role of Fas in triggering hepatocyte death, we developed a tissue and gene-specific silencing approach using targeted nanoparticles bearing siRNA, which was capable of successfully targeting hepatocytes, inducing gene silencing, and inhibiting pathological changes associated with the hepatotoxic process.

The major challenge in development of siRNA therapeutics is delivery methods to the target cells in vivo. This is caused in part due to poor intracellular uptake and limited blood stability of siRNA. In order to achieve hepatocyte-targeted delivery of Fas siRNA and improved gene transfer by intravenous injection of a liposomes complex, some modifications were made to the original formulation to increase specifically targeting hepatocytes and encapsulation efficiency.

On the basis of previous reported that the galactose is a ligand that binds the asialoglycoprotein receptors (ASGP-R), which is specific for hepatocytes with a high affinity and a rapid internalization rate [23], galactosylated lipid was chemically synthesized through link galactose to the distal end of DSPE-PEG. DSPE-PEG is a PEGlyated lipid, was prepared to keep the stabilization of the particles in a physiological condition and prolong the circulation time in vivo [23], [28]. Cationic liposomes tend to aggregate in the presence of serum proteins and are taken up by the reticuloendothelial system due to their positive charge content [29]. To increase the serum stability of liposomes, the liposome surface was decorated with 10 mol % Gal-DSPE-PEG lipids by the post-insert method, which assures target ligand localization on the external surface of liposomes. Although some researchers reported ability to increase DSPE-PEG2000 concentration to 20 mol % to preventing aggregation induced by serum proteins [24], in our new liposome formulation, we found when increase DSPE-PEG2000 to 20 mol %, the zeta potential of liposomes become negative charged and reduce transfection efficiency is observed.

Protamine is a highly positive charged peptide that acts as nucleic acid condensation reagent. It reported that condensed siRNAs with protamine can increase delivery efficiency [30]. Apart from this, we found when lipid film was hydrated with a solution composed of protamine and condensed siRNA, it can achieves more than 90% entrapment efficacy compare with our previous report [31]. Correspondingly, the encapsulated siRNA was also sufficient for silencing Fas gene expression.

To develop efficacy and safety hepatocyte targeting siRNA delivery system, the side effect of immune activation with excessive cytokine release and associated inflammatory syndromes should be avoided. Therefore, we tested the levels of IFN-γ, TNF-α as well as IL-6 to investigate the toxicity of the Gal-LipoNP/siRNA complex. As shown in Figure 5, none of the Gal-LipoNP/siRNA complexes causes significant inflammatory syndromes and hepatitis in mice.

We employed two different delivery approaches to detect the gene silence efficiency. High-volume intravenous (hydrodynamic) injection of naked siRNA is currently the most common method for inducing RNAi in laboratory animals, despite the global gene-silencing effects and lack of feasibility for human application [32], [33]. Although Song et al reported that the use of naked Fas siRNA through hydrodynamic injection can protect mice from fulminant hepatitis [9], this treatment required three times administration of Fas siRNA before ConA-induced hepatitis, which may limit therapeutic attractiveness. Water et al [34] investigated the behavior of naked siRNA distribution in vivo and reported that naked siRNA preferentially accumulates in the kidneys and is excreted in the urine. Santel et al [35] also reported the biodistribution peculiarity of naked Cy3 labeled siRNAs and discovered after 20 min post-injection, the naked siRNAs are predominantly found in the kidney, with no detectable signals in heart, liver, spleen, lung, and pancreas. Furthermore, they reported that naked siRNAs accumulates in the pole and lumen of the proximal tubules and in the urine 5 min after intravenous injection. These studies revealed that there is no selective uptake of siRNA to any cell type of the tissues investigated in vivo, and this is most likely due to instant renal excretion. Consistent with the current literature, we found that the majority of intravenously injected naked siRNA was concentrated in and cleared by the kidney soon after administration (Figure 2). If naked Fas siRNA was injected for one time and only less than 20% gene silence efficiency can be observed. In contrast, Gal-liposomes were primarily found in the liver at early time points and accumulated in liver until 48 h later can still see red fluorescence. Thus, our results suggest that Gal-liposomes mediated in vivo delivery may endow siRNA with a time-release property. Because siRNA is protected from endogenous nucleases within the liposomes interior and was targeted to hepatocytes through a specific ligands, it is possible that Gal-LipoNP accumulated in the hepatocytes-populated areas of the liver and slowly released their protected siRNA cargo, resulting in a prolonged gene-silencing effect.

In our study significant therapeutic effects were obtained in ConA induced hepatitis by specific silence Fas gene. Fas-mediated apoptosis play an important role in viral and autoimmune hepatitis, alcoholic liver disease [36]. The present study focuses on hepatocyte-targeted silencing of Fas gene. The use of Gal-LipoNP and hepatocyte-targeted delivery of siRNA is not only applicable for fulminant hepatitis but also for many other liver diseases such as transplant rejection, hypercholesteremia, and hepatic genetic diseases. Similar strategy can be used to knock down other critical molecules associated with liver fibrosis, inflammation, as well as liver viral infections, such as hepatitis B virus [37] or hepatitis C virus [38].

In summary, our study demonstrates that siRNA could be specific delivered to the liver using galactosylated liposomes. The Gal-liposomes/siRNA complex can efficiently knock down Fas gene expression in liver; the results further clearly demonstrate that the application of Fas siRNA can prevent fulminant hepatitis. Consequently, the use of galactose ligated liposomes delivering siRNA may represent a promising clinical application of RNAi as a powerful tool for molecular therapy of many liver diseases.
